# Supplementary material for: Patterns of Intron Gain and Loss in Fungi
Source: PLoS Biol. 2004 Nov 30;2(12):e422. doi: 10.1371/journal.pbio.0020422 (PMC532390; doi:10.1371/journal.pbio.0020422)
Supplement: Table S1 — Also available at http://genes.mit.edu/NielsenEtAl/. (4.3 MB ZIP). [file pbio.0020422.st001.zip › NielsenEtAl/html/722.html]

AN4702.1.NCU00881.1.MG10586.1.FG02810.1


```
 CLUSTAL W (1.82) Multiple Sequence Alignments - Introns Inserted


Sequence 1: NCU00881.1	229 aa
Sequence 2: MG10586.1	233 aa
Sequence 3: FG02810.1	215 aa
Sequence 4: AN4702.1	244 aa
Alignment Length: 260 aa
Number Identitical Residues: 83 aa
Alignment Score (without introns) 4900


MG10586.1 	MLPST------ILVLASAALSTAHTVITYPGWRGDNLVT-----------------NET-
NCU00881.1	MLTST------LLALASAALASAHTVITYPGWRGNNLKD-----------------NEE-
FG02810.1 	MRTSA------LLTFVLATIVSAHSVITYPGWRGNNLIT-----------------NAT-
AN4702.1  	MLMSTSPSPWLAAAMLCIGLANAHTVIVYPGYRGNNLHTTGTVEGADGLGIAWSSDNETL
          	*  *::.:.    .:    : .**:**.***:**:**  :.: ..:.. . : ::.*   

MG10586.1 	-YPYGMQWIYPC~GGINLTQNRTYWSTKGGAVALQPGWFQGHQTATLYVNLGIGNKGPDG
NCU00881.1	-FPYGMQWTYPC1GGLTLTQNRTYWPTTGGAISFQPGWFQGHATAFIYVNLGIGNDGPDG
FG02810.1 	-FPYGMQWIYPC1G----------------AVAFQPGWFRGHETAFAYINMGFGDDGPDG
AN4702.1  	IYPYGMQWSYPC1GGMPTSENRTKWPVQGGAVSFQPGWFQGHSRAQIYINIGLGTVPP--
          	 :****** *** *.   :.. :  .  ..*:::*****:**  *  *:*:*:*   *  

MG10586.1 	GPLNMSLSMVPAFGISGPSKNPYPGTICLPQVSLPQGYTPEVGENATIQVVELAVHGAAL
NCU00881.1	GPKNMSFPMVSPFQIIGPGKNPYPGTFCLPQVSTPAGFEFKEGDNATIQLVELAIHGAAL
FG02810.1 	GPANMTLPMIPMFQILGPTNNPFPGTICLPQVPLPKNTTVKAGDTATIQVVELAIHGAAL
AN4702.1  	---NMSHPMITPFEIVGPDNDPYPGTICLPQVRLPAGIEVQVGDNATIQVIELAQHGAAL
          	   **: .*:. * * ** ::*:***:*****  * .   : *:.****::*** *****

MG10586.1 	FS0CVDITFVEPDDERLEKVHADNCFNSTDIGFTDIYTTKVANTNITSTSAAAASLRQPG
NCU00881.1	YS0CVDITFVPPGDPRVAQVNESNCFNSTDLGAADLYTINILESGRDRLALTSAAAS---
FG02810.1 	YS0CVDIIFADPGDKRLKEVNETNCFNSTDIGFADIYTITTKESGSDEYASESGATP---
AN4702.1  	YN0CADITFAEPED--VAEVNSTNCFNSSHISFETIFTTSSLEN----AGLEAVTVP---
          	:. *.** *. * *  : :*:  *****:.:.   ::* .  :.     .  : :     

MG10586.1 	GLAWYAGWIPMVGLGAWALL--
NCU00881.1	-LARMAGWVPLVAGGLWLML--
FG02810.1 	-AFRQLGWIPLVAAGLWAFLLA
AN4702.1  	--SFLTAVVPTFLGIAYGLLMA
          	      . :* .    : :* :
```
